# Thromboelastography in the Perioperative Period: A Literature Review

**DOI:** 10.7759/cureus.39407

**Published:** 2023-05-23

**Authors:** Vendhan Ramanujam, Stephen DiMaria, Vivek Varma

**Affiliations:** 1 Department of Anesthesiology, Rhode Island Hospital/The Warren Alpert Medical School of Brown University, Providence, USA

**Keywords:** outcomes, coagulation, transfusion, perioperative period, teg, thromboelastography

## Abstract

Assessing coagulation status is essential for prompt intervention to reduce morbidity and mortality related to bleeding and thrombotic complications during the perioperative period. Traditional coagulation tests such as platelet count, activated partial thromboplastin time (aPTT), prothrombin time (PT), international normalized ratio (INR), and activated clotting time (ACT) provide only static evaluation. These tests are not designed for assessment of dynamically changing coagulation conditions during the perioperative time. However, viscoelastic coagulation testing such as thromboelastography (TEG) produces a rapid numerical and graphical representation that helps to detect and direct targeted hemostatic therapy. Searching the literature through PubMed, Medline, Ovid, CINAHL, and ClinicalTrials.gov we retrieved 210 studies, which represent the use of TEG in the perioperative period. The included studies were categorized under various settings such as trauma, obstetrics, orthopedics, intensive care unit (ICU), cardiovascular, transplant, and miscellaneous scenarios. TEG showed promising results in trauma surgeries in predicting mortality, hypercoagulability, and bleeding even when it was compared to conventional methods. TEG was also useful in monitoring anticoagulant therapy in orthopedic and obstetric surgeries; however, its role in predicting thrombotic events, hypercoagulability, or complications was questionable. In ICU patients, it showed promising results, especially in the prediction or improvement of sepsis, coagulopathy, thrombotic events, ICU duration, hospital stay, and ventilator duration. TEG parameters effectively predicted hypercoagulation in transplant surgeries. Regarding cardiovascular surgeries, they were effective in the prediction of the need for blood products, coagulopathy, thrombotic events, and monitoring anticoagulation therapy. More randomized clinical trials comparing TEG parameters with standardized tools are needed to produce robust results to standardize its use in different perioperative settings.

## Introduction and background

Monitoring of blood coagulation status during the perioperative period is crucial for prompt intervention as bleeding and thrombotic complications related to surgery can significantly affect morbidity and mortality. Assessing the coagulation status comprehensively is a challenge since the coagulation cascade is dynamic and depends on the interaction of several factors including primary hemostasis, platelet clot formation, secondary hemostasis, thrombin generation, and fibrinolysis [1]. Traditional coagulation tests such as platelet count, activated partial thromboplastin time (aPTT), prothrombin time (PT), international normalized ratio (INR), activated clotting time (ACT), and plasma fibrinogen levels provide only static evaluation of the patient and are not designed for assessment of dynamically changing coagulation conditions during perioperative time; thus, they lack the ability to direct targeted hemostatic therapy [2]. However, viscoelastic coagulation testing such as thromboelastography (TEG) is devised for a quick global assessment of hemostasis more like in vivo hemostasis by continuously monitoring the clotting process from its steps of initiation, amplification, propagation, and termination through fibrinolysis. They produce a rapid numerical and graphical representation that helps clinicians with the early management of goal-directed hemostatic resuscitation and anticoagulation effects [3-6]. Our goal is to systemically search and summarize the existing evidence from studies that have reported the utility of viscoelastic coagulation testing and its impact on clinical outcomes during the perioperative period.

Thromboelastography (TEG)

TEG is a whole blood-based assay that runs at 37°C to mimic natural blood clotting in vivo [7]. The instrument consists of a pin immersed into a cup containing whole blood that begins to clot when a constant rotational force is applied to it. As the viscosity of blood increases, the pin becomes cross-linked to the cup via fibrin and platelet interactions. Now there is a torque between the cup and the pin, and the movement of the pin produces an electrical signal that is traced as a curve over time. As the clot breaks down and torque decreases, the tracing fades. The signals are then interpreted by TEG software where changes in amplitude are plotted, and different parameters of the curve are measured to assess coagulation status [8]. The parameters include reaction (R) time, coagulation (K) time, alpha (α) angle, maximum amplitude (MA), and lysis at 30 minutes (LY30). The tracing and results are available in real-time, enabling prompt interpretation for goal-directed therapy [9]. While TEG is favored in North America, there are other viscoelastic tests such as rotational thromboelastometry (ROTEM) that are favored in Europe. Both the tests are equivalent with interchangeable results and interpretations, yet characteristics and nomenclature differences exist, and they are illustrated in Tables 1-2.

**Table 1:** Characteristic differences between TEG and ROTEM TEG: thromboelastography; ROTEM: rotational thromboelastometry

Characteristic	TEG	ROTEM
Cup motion	Moving	Fixed
Pin motion	Fixed	Moving
Pipetting	Manual	Automated
Detector system	Torsion Wire	Optical
Samples ran at one time	Two	Four

**Table 2:** Nomenclature differences between TEG and ROTEM TEG: thromboelastography; ROTEM: rotational thromboelastometry

TEG	ROTEM
Reaction time (R-time)	Clotting time (CT)
Coagulation time (K-time)	Clot formation time (CFT)
Maximum amplitude (MA)	Maximum clot firmness (MCF)
Lysis 30 (LY30)	Lysis Index 30 (LI30)

Interpretation of parameters

Reaction Time (R-Time)

Reaction time is the first measurement of the coagulation cascade. Its measurement is related to coagulation factor activation. This value is similar to extrinsic and intrinsic clotting pathway measurements by PT and aPTT respectively. The R-time largely reflects the adequacy of coagulation factors and is the most sensitive parameter to measure the effects of heparin therapy including low molecular weight heparin (LMWH) [10, 11]. An elevated or prolonged R-value (more than eight minutes) can signify a deficiency in clotting factors, hemodilution, or the presence of heparin. Therefore, indicating a need for transfusion of fresh frozen plasma. On the other hand, a shortened R-time (less than four minutes) can indicate hypercoagulopathy requiring the use of anticoagulation.

K-Time

It is a measurement of the time interval between R and time to reach 20 mm clot amplitude. K-time and α angle are both related to coagulation factor amplification. Therefore, their values correlate, and they both indicate a deficiency in clot growth kinetics. A low value can indicate a deficiency in fibrinogen and may reveal a need for cryoprecipitate. A high value is similar to the R-time, which represents a hypercoagulable state, and an anticoagulant may be required.

α Angle

It is a measurement of the line tangent to the slope of the curve during clot formation. The computer software calculates the angle based on the slope and time. A number of factors including thrombin generation and fibrinogen levels determine the angle. It identifies states of hyper- or hypo-coagulopathies.

MA Value

It is the maximum amplitude that represents the distance traveled by the cross-linked cup/pin. It is a measurement of maximum clot strength and provides information on both fibrinogen and platelet function. A high MA value may indicate hypercoagulation and the need for an anticoagulant. A low MA value indicates low clot strength, which can be caused by decreased fibrinogen levels, low platelet counts, or decreased platelet function. If a low MA is combined with a decreased K value, this is an indication of cryoprecipitate therapy. MA value is very important when paired with a platelet count because a low platelet count and a normal MA value indicate a patient has a normal platelet function and therefore does not require platelet transfusion. Conversely, treatment with platelets may be indicated for patients with a low MA value, low platelet function, and normal platelet count [12]. 

LY30

It is clot lysis at 30 minutes. It is the last major TEG parameter and measures the percent of the decrease in area under the curve over 30 minutes. Therefore, it reflects fibrinolysis after maximum amplitude is reached. This measurement is most useful for patients undergoing thrombolytic therapy or during disseminated intravascular coagulation (DIC). A high LY30 percentage indicates hyperfibrinolysis and patients may require antifibrinolytic agents including tranexamic acid, aprotinin, and aminocaproic acid.

## Review

Methods

A review of the literature was conducted to identify qualifying publications. The search was conducted in the following databases: PubMed, Medline, Ovid, CINAHL, and ClinicalTrials.gov. Search criteria were defined using the string (thromboelastography or TEG) and (perioperative or postoperative or preoperative or operative) in all search fields. Inclusion criteria for the systematic review included articles that represented original research including as a focal outcome evaluation of TEG procedures; in one or more perioperative settings (pre, intra, or postoperative); in a human population; have been published in a peer-reviewed source and in English. Excluded items included theses or dissertations, conference abstracts, and proceedings, theoretical papers, comments or letters to the editor, or previous reviews. We abstracted data from selected studies that include patient samples, perioperative settings where TEG was utilized, TEG parameters that were assessed, and clinical outcomes that were reported. Because of clinical and methodologic heterogeneity among studies, we expected to report results qualitatively rather than conducting a meta-analysis.

**Figure 1:**
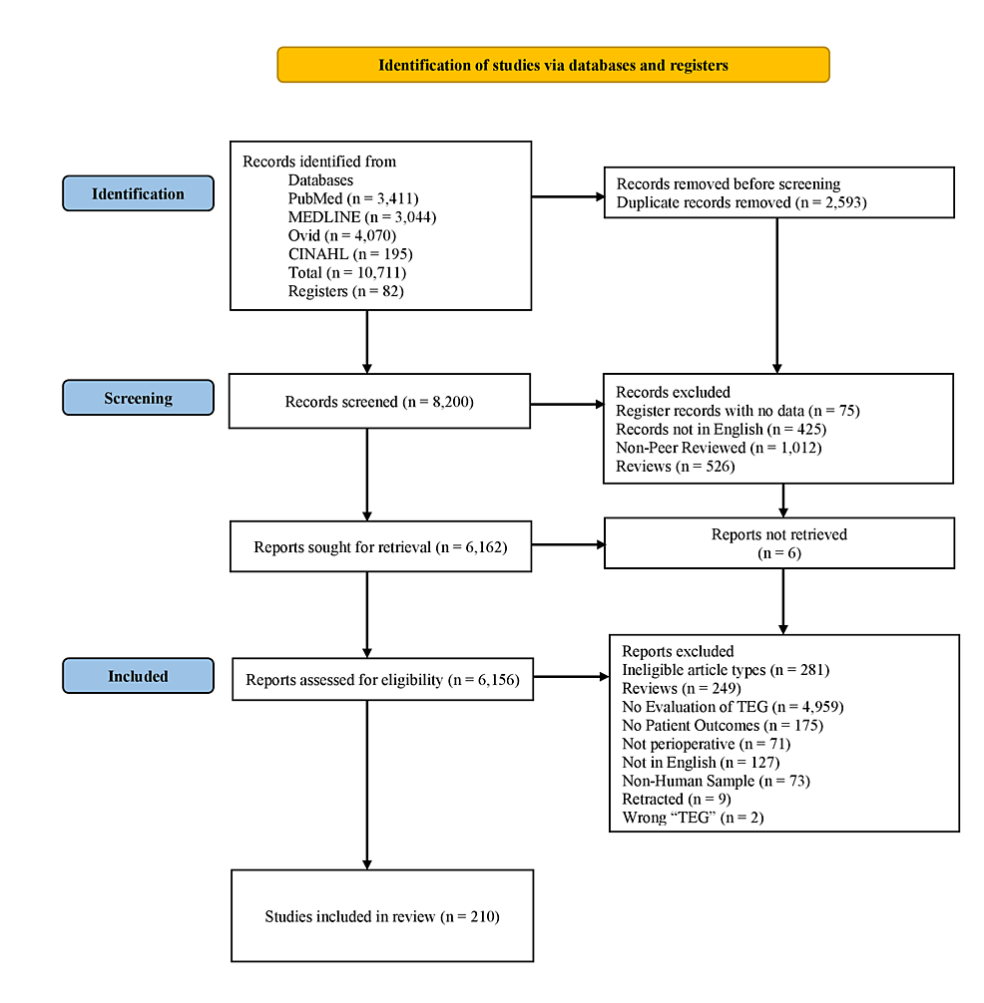
PRISMA flow diagram with included searches of databases and registers PRISMA: Preferred Reporting Items for Systematic Reviews and Meta-Analyses

The initial database search described in the methods section yielded 8,200 unique articles after duplicates were removed. Among them, 6,156 reports were included and assessed for eligibility after excluding records without data (N = 75), not in English (N = 425), that were non-peer-reviewed (e.g., conference abstracts) (N = 1,012), that were reviewed (N = 526) and that were not retrievable (N = 6). After further automated and manual screening of assessed reports for eligibility, 210 articles were found to be eligible and included in the review (Figure 1). Reasons for rejection of assessed articles included studies that were ineligible (N = 281), were reviews (N = 249), in which TEG was mentioned but not evaluated (N = 4,959), did not include patient outcomes (N=175), studies not in perioperative settings (N = 71). Also, articles in languages other than English (N = 127), studies with veterinary samples (N = 73), retracted studies (N = 9), and the use of the abbreviation TEG not referring to thromboelastography (N = 2) were excluded.

The included 210 studies were categorized under various surgical settings. Studies in the cardiovascular settings were the maximum with 64 studies while those based in the surgical intensive care unit (ICU) setting were the least with only one study.

Trauma

TEG finds its clinical application critically useful in trauma; the American College of Surgeons recommends it to be available at all level I and level II trauma centers. Complications from trauma-related surgery such as hemorrhage and thrombosis remain the leading causes of preventable death. Hemorrhage exacerbation is associated with trauma-induced coagulopathy (TIC) and has been shown to be present in more than 25% of severely injured patients upon arrival to the emergency department. TIC is a lethal, unbalanced, and abnormal process. Its early stages are characterized by hypercoagulability and bleeding whereas the later stages are characterized by hypercoagulability with venous thromboembolism and multiple organ failure. In such a scenario, comprehensive information about coagulation status is essential. TEG by analyzing various contributors of both hemostasis and clot dissolution provide extensive information that has been shown to predict mortality as well as positively impact it during TIC [13-15]. TEG parameters such as MA and R-time detect platelet function and coagulation factor deficiency with a high degree of specificity that guide individualized therapy for patients [16, 17]. It is accurate in diagnosing hypofibrinogenemia as well [18]. Their ability to reliably detail the hypercoagulable states in cancer patients and the distorted coagulation status in alcoholic patients during trauma is well demonstrated [19, 20]. Overall, they reflect coagulation status better than traditional coagulation tests [21].

Since TIC is associated with uncontrolled bleeding, TEG's ability to provide insight into both depletion coagulopathy and hyperfibrinolysis allows it to guide massive transfusion protocol (MTP); and predict the associated mortality [22-25]. TEG-guided resuscitation has demonstrated lower blood product usage, shorter ICU and hospital stay, and lower overall costs especially when compared to conventional coagulation tests that come with limitations such as its time-consuming nature, failure to delineate the complex nature of TIC, and unclear value in guiding transfusion [26-29]. This has been shown to improve mortality outcomes, especially in pelvic fractures, penetrating as well as blunt trauma patients, and burn patients [30-33]. While the ability of TEG to predict transfusion has been replicated in the general population it was not the case in the polytrauma population [34]. Using TEG protocols that are directed to reduce blood product usage and improve survival [35, 36], transfusion has become more patient-specific with an average transfusion ratio of 2.5:1:2.9 (red blood cells: plasma: platelets), different from the current 1:1:1 guideline [37]. TEG has been shown to be valid during MTP and results in different patterns of blood transfusion based on individual patient requirements as well as a reduction in overall hemorrhage-related deaths during trauma [38, 39]. When it comes to MTP-related blood product usage, TEG does not differ from conventional testing and ROTEM [40, 41]. During trauma surgery involving the liver and spleen, interestingly, TEG guidance has demonstrated less as well as increased blood product usage but shorter surgery time [42, 43]. With the success of TEG in assessing coagulopathic parameters in trauma patients, TEG has been investigated for detecting and reversing anticoagulants only with limited success, and conventional tests (e.g., PT, INR, PTT) that have shown better results comparatively have been recommended [44-47]. TEG finds its utility in the pediatric trauma population as well where it has been shown to accurately predict MTP requirement, thromboembolism, and mortality [48, 49, while outcomes such as blood product use, ventilator duration, and length of ICU stay were found to be worse with TEG use there was no change in mortality [50] (Table 3).

**Table 3:** Studies of TEG in trauma perioperative settings TEG: thromboelastography; TEG-MA: TEG with maximum amplitude; rTEG; rapid thromboelastography; tPA-TEG: tissue plasminogen activator; TEG-PM: TEG with platelet mapping; TBI: traumatic brain injury; VTE: venous thromboembolism; ROTEM: rotational thromboelastometry; MTP: massive transfusion protocol

Citation	Patient Sample	Operative Setting	TEG Procedures Assessed	Clinical Outcomes	Summary of Findings
Farrell et al., 2021 [13]	50 US trauma patients	Intraoperative	TEG-MA, TEG R-time, TEG-K, TEG alpha-angle	Mortality	“Death diamond” combination of TEG parameters is strongly predictive of mortality after trauma
Chin et al., 2014 [14]	98 US trauma patients	Intraoperative	rTEG-MA, rTEG R-time, rTEG-K, rTEG alpha-angle, rTEG-LY30	Mortality, coagulopathy	rTEG parameters predicted coagulopathy, coagulopathy impacted mortality differently among different subsets of patients
Albert et al., 2019 [15]	58 Indian trauma patients with TBI	Intraoperative prior to transfusion	TEG R-time, k-time, alpha-angle	Coagulopathy	TEG values including prolonged k-time and shortened alpha angle predicted coagulopathy after TBI
Moore et al., 2015 [16]	58 US trauma patients	Intraoperative	TEG-MA	Platelet function	TEG-MA was predictive of platelet function
Chow et al., 2020 [17]	550 US trauma patients	Intraoperative	TEG R-time	Coagulation factor deficiency	TEG R-time predicts coagulation factor deficiency with high specificity but low sensitivity
Chow et al., 2019 [18]	623 US trauma patients	Intraoperative	TEG-MA, TEG-K, TEG alpha-angle	Hypofibrinogenemia	TEG parameters predict hypofibrinogenemia with high specificity but low sensitivity
Mou et al., 2019 [19]	157 Chinese oncology patients	Preoperative	TEG-MA, TEG R-time, TEG-K, TEG alpha-angle	Hypercoagulability, VTE, thrombotic complications	TEG parameters were related to hypercoagulability, but not to VTE or thrombosis
Howard et al., 2014 [20]	415 US trauma patients	Intraoperative	TEG-MA, TEG R-time, TEG-K, TEG alpha-angle	Hypocoagulation in relation to alcohol exposure	TEG parameters gave inaccurate indications of hypocoagubility in patients with alcohol exposure
Liu et al., 2016 [21]	40 Chinese older adult fracture patients	Preoperative	TEG-MA, TEG R-time, TEG-K, TEG alpha-angle	Coagulopathy	TEG parameters predicted coagulopathy more accurately than traditional lab values
Ives et al., 2012 [22]	118 US trauma patients	Preoperative	TEG-MA, TEG R-time, TEG-K, TEG alpha-angle	Coagulopathy, MTP, mortality	TEG parameters predicted MTP and mortality
Coleman et al., 2018 [23]	343 US trauma patients	Intraoperative	TEG-MA, TEG R-time, TEG-K, TEG alpha-angle	Massive transfusion status	TEG parameters are predictive of the need for massive transfusion
Moore et al., 2017 [24]	324 US trauma patients	Intraoperative	rTEG tPA–TEG	MTP	tPA-TEG parameters efficiently identify patients needing MTP
Pezold et al., 2012 [25]	80 US trauma patients	Intraoperative	TEG-MA, TEG R-time, TEG-K, TEG alpha-angle	Mortality	TEG parameters predict early mortality
Mohamed et al., 2017 [26]	134 US trauma patients	Intraoperative	TEG-guided resuscitation protocol vs. clinician discretion	Blood product usage, ICU length of stay, hospital length of stay, costs	Introduction of TEG-guided resuscitation protocol resulted in lower blood product usage, shorter ICU length of stay, shorter hospital length of stay, and lower overall costs
Holcomb et al., 2012 [27]	1,974 US trauma patients	Intraoperative	TEG-MA, TEG R-time, TEG-K, TEG alpha-angle	Blood product usage	TEG parameters were superior predictors of blood product usage compared with conventional coagulation tests
Kaufmann et al., 1997 [28]	69 US blunt trauma patients	Intraoperative	TEG-MA, TEG R-time, TEG-K, TEG alpha-angle	Hypercoagulability, transfusion	TEG parameters were predictive of need for transfusion
Schochl et al., 2010 [29]	681 Austrian trauma patients	Intraoperative	TEG-guided hemostatic therapy vs. clinician discretion	Blood product usage	TEG-guided protocol resulted in lower blood product usage volume
Kane et al., 2015 [30]	131 US pelvic trauma patients	May vary (retrospective record review)	TEG R-time	Mortality	TEG R-time was predictive of mortality risk
Bostian et al., 2020 [31]	141 US trauma patients	Preoperative	TEG-LY30	Mortality, blood loss, transfusion, hemoglobin changes	TEG parameters on intake were associated with extent of blood loss, volume of blood products transfused, and mortality risk
Tapia et al., 2013 [32]	289 US trauma patients	Intraoperative	TEG-directed resuscitation protocol vs. standardized MTP protocol	Blood product volume, mortality	TEG-directed resuscitation had better mortality outcomes for penetrating trauma, and lower blood product usage volume for more severe blunt trauma patients compared with standardized MTP protocol
Huzar et al., 2018 [33]	65 US burn patients	May vary (retrospective registry study)	TEG-MA, TEG R-time, TEG-K, TEG alpha-angle, TEG-LY30	Resuscitation, transfusion volumes, mortality	TEG parameters predicted resuscitation, transfusion volumes, and mortality
Van Wessem and Leenen, 2017 [34]	135 Dutch trauma patients	Intraoperative	TEG-MA, TEG R-time, TEG-K, TEG alpha-angle	Coagulopathy	TEG parameters were not predictive of coagulopathy
Gonzalez et al., 2016 [35]	111 US trauma patients	Intraoperative	TEG-directed MTP protocol vs. conventional MTP protocol (RCT)	Survival, blood product volume	The TEG-directed protocol increased survival and reduced blood product usage
Sumislawski et al., 2018 [36]	278 US trauma patients	Intraoperative	TEG-guided resuscitation vs. conventional assay-guided resuscitation	Mortality, coagulopathy	Patients treated with TEG-guided protocols had better survival
Mamczak et al., 2016 [37]	40 US trauma patients	Intraoperative	TEG-PM guided transfusion protocol	Blood product usage	TEG-guided transfusion protocol resulted in different patterns of blood product usage from standardized modality
Stettler et al., 2018 [38]	825 US trauma patients	Intraoperative	TEG-MA, TEG R-time, TEG-K, TEG alpha-angle, TEG-LY30	MTP administration	TEG parameters are valid for use in guiding MTP administration
Johansson et al., 2013 [39]	182 Danish trauma patients	Intraoperative	TEG-MA, TEG R-time, TEG-K, TEG alpha-angle	Survival, transfusion, blood product volume	TEG parameters differed between survivors and non-survivors but did not independently predict survival
Unruh et al., 2019 [40]	67 US trauma patients	Intraoperative	TEG-guided MTP vs. conventional testing	Blood product usage	There was no difference in blood product usage between TEG-guided MTP and conventional testing-guided MTP
Rizoli et al., 2016 [41]	33 Canadian trauma patients	Intraoperative	TEG vs. ROTEM parameters in comparison	Coagulopathy	TEG and ROTEM parameters had similar performance for detecting intraoperative coagulopathy
Dudek et al., 2021 [42]	258 US trauma patients	Intraoperative	TEG-guided transfusion vs. standardized MTP	Volume of blood product use, time to surgery	Patients receiving TEG-guided transfusion received more blood products and had a shorter time to surgery
Wang et al., 2017 [43]	166 US trauma patients	Intraoperative liver or spleen surgery	TEG-guided blood component therapy vs. clinician discretion	Blood product usage, hospital length of stay	TEG-guided therapy was associated with lower blood product usage volume and shorter average hospital length of stay
Barton et al., 2021 [44]	824 US trauma patients	May vary (retrospective observational study)	TEG-PM	Preoperative anticoagulation	TEG parameters can differentiate some but not all common anticoagulants. Authors recommend investigation of other methods detecting need for anticoagulation reversal
Kobayashi et al., 2018 [45]	182 US trauma patients	Preoperative	TEG-MA, TEG R-time, TEG-K, TEG alpha-angle	Coagulopathy due to novel oral anticoagulant (NOA) therapy	TEG parameters were not effective at detecting coagulopathy due to NOA therapy
Ali et al., 2017 [46]	54 US trauma patients	Intraoperative	TEG-MA, TEG R-time, TEG-K, TEG alpha-angle	Preoperative anticoagulation, postoperative coagulopathy	TEG did not differentiate patients with preoperative anticoagulation therapy. Authors recommend using conventional testing methods to identify patients in need of anticoagulation reversal
Myers et al., 2020 [47]	100 US trauma patients	Intraoperative	TEG-MA, TEG R-time, TEG-K, TEG alpha-angle	Anticoagulation reversal detection	TEG parameters correlate with anticoagulation reversal, but conventional tests perform better in clinical settings
Leeper et al., 2018 [48]	133 US pediatric trauma patients	Preoperative	TEG-MA, TEG R-time, TEG-K, TEG alpha-angle	Coagulopathy, thromboembolism, transfusion, mortality	TEG parameters can be combined with other variables as part of a principal components analysis to predict transfusion, thromboembolism, and mortality
Phillips et al., 2021 [49]	117 US pediatric trauma patients	Intraoperative	TEG-MA, TEG R-time, TEG-K, TEG alpha-angle	MTP implementation	TEG parameters accurately identify patients needing MTP
Aladegbami et al., 2018 [50]	125 US pediatric trauma patients	Intraoperative	rTEG vs. other non-TEG assessments (observational study)	Mortality, blood product use, ventilator duration, length of ICU stay	Patients with rTEG had worse outcomes on all measures except mortality (which did not differ). However, rTEG was used primarily for more severely injured patients

Obstetric

Pregnancy is uniquely a hypercoagulable state. This usually results in thromboembolic complications that can affect the pregnancy, necessitating the need for anticoagulants. Under such circumstances, TEG parameters have been found to be useful in guiding anticoagulation therapy [51]. But its sensitivity has not been found to be adequate to monitor the progress of anticoagulation [10]. On the other hand, pregnancy-related complications such as pre-eclampsia and eclampsia reverse the blood coagulability into the hypercoagulable state as well as hemolysis that can be exacerbated during surgery. Although TEG relates such coagulopathic scenarios during pregnancy with the risk profiles preoperatively [52], they have yet been found to be inferior when compared to conventional coagulation tests in predicting intraoperative coagulopathy and blood loss [53]. They still have been demonstrated to reduce blood product use, costs, risks of ICU admission, and the need for emergency hysterectomy [54] (Table 4).

**Table 4:** Studies of TEG in obstetric perioperative settings TEG: thromboelastography; TEG-MA: TEG with maximum amplitude

Citation	Patient Sample	Operative Setting	TEG Procedures Assessed	Clinical Outcomes	Summary of Findings
Griffiths et al., 2017 [10]	24 UK obstetric patients	Postoperative Cesarean section	TEG-MA, TEG R-time, TEG-K, TEG alpha-angle	Anticoagulant detection	TEG parameters do not have the sensitivity to accurately monitor anticoagulant therapy progress
Boyce et al., 2011 [51]	19 UK obstetric patients	Intraoperative Cesarean section	TEG R-time	Response to heparin dosage on coagulation	TEG parameters were useful for guiding heparin dosage
Karlsson et al., 2014 [52]	45 Swedish obstetric patients	Intraoperative	TEG-MA, TEG R-time, TEG-K, TEG alpha-angle	Coagulopathy, blood loss	TEG parameters were worse predictors of coagulopathy and blood loss compared with conventional laboratory tests
Smith et al., 2009 [53]	54 UK obstetric patients	Preoperative, postoperative Caesarian section	TEG-MA, TEG R-time	Coagulopathy, risk profiles	TEG parameters were associated with postoperative coagulopathy and risk profiles
Snegovskikh et al., 2018 [54]	86 US obstetric patients	Preoperative transfusion due to severe hemorrhage	TEG-directed transfusion protocol vs. clinician discretion	Blood loss, blood product use, ICU admission, emergency hysterectomy, costs	Introduction of TEG-directed transfusion protocol reduced blood product use, costs, and risks of ICU admission or emergency hysterectomy

Orthopedic

Orthopedic surgery in general involves the release of massive tissue factors triggering a coagulation process that requires anticoagulants for venous thromboembolism prevention and treatment. For joint surgeries, neuraxial and peripheral nerve blocks are mainstay anesthesia choices that need information on the patient’s coagulation profile and medications that affect coagulation. So comprehensive information about coagulation status in orthopedic surgery patients is important. In demographic-specific orthopedic surgery patients, TEG has been reported to be a better measure of hypercoagulability compared to conventional measures [55] but has been found not to predict venous thromboembolism risk [56]. In spine surgery, TEG has predicted clotting factor deficiency such as hypofibrinogenemia and was found to be an inferior predictor of coagulation status as a whole compared to traditional laboratory measures [57, 58]. However, their sensitivity to sustained coagulation changes i.e., after seven days is superior compared with traditional measures [59]. When it comes to anticoagulation, TEG has been established to differentiate anticoagulated patients as well as monitor their therapy [60-62]. TEG-guided anticoagulation prophylaxis has better safety and comparable efficacy to conventional prophylaxis strategy [63]. TEG did not find any significance in detecting specific outcomes related to orthopedic surgery such as bone cement implantation syndrome and infections [64, 65] (Table 5).

**Table 5:** Studies of TEG in orthopedic perioperative settings TEG: thromboelastography; TEG-PM: TEG with platelet mapping; TEG-MA: TEG with maximum amplitude; VTE: Venous thromboembolism

Citation	Patient Sample	Operative Setting	TEG Procedures Assessed	Clinical Outcomes	Summary of Findings
Lloyd-Donald et al., 2021 [55]	52 Australian orthopedic patients	Preoperative, intraoperative, postoperative	TEG-MA, TEG R-time, TEG-K, TEG alpha-angle	Hypercoagulability	TEG parameters were a better measure of hypercoagulability in this population than conventional measures
Parameswaran et al., 2016 [56]	101 Indian orthopedic patients	Preoperative	TEG-MA, TEG R-time, TEG-K, TEG alpha-angle	VTE	TEG parameters did not predict VTE risk
Horlocker et al., 2001 [57]	244 US spinal surgery patients	Intraoperative spinal fusion	TEG-MA, TEG R-time, TEG-K, TEG alpha-angle	Coagulopathy	TEG parameters were worse predictors of coagulation status than traditional laboratory measures
Chen, Hu, et al., 2020 [58]	39 Chinese adolescent orthopedic patients	Intraoperative scoliosis surgery	TEG-FLEV	Hypofibrinogenemia	TEG-FLEV predicts hypofibrinogenemia
Bai et al., 2021 [59]	228 Chinese orthopedic patients	Pre- and post-operative THA	TEG R-time, TEG-MA	Coagulability after anticoagulant prophylaxis (1 and 7 days post-surgery)	TEG R-time and TEG-MA measures were both more sensitive to sustained coagulation changes after 7 days compared with traditional laboratory measures
Klein et al., 2000 [60]	24 US orthopedic patients	Preoperative, postoperative	TEG-MA, TEG R-time, TEG-K, TEG alpha-angle	Anticoagulation	TEG parameters differentiate anticoagulated patients
Li et al., 2020 [61]	80 Chinese orthopedic patients	Intraoperative posterior lumbar fusion	TEG-MA, TEG R-time, TEG-K, TEG alpha-angle, TEG-CI, TEG-PIR	Anticoagulation monitoring	TEG parameters are useful for monitoring anticoagulant therapy
Tekkesin et al., 2016 [62]	30 Turkish orthopedic patients	Preoperative, postoperative	TEG R-time	Anticoagulation monitoring	TEG parameters are useful for monitoring anticoagulation therapy perioperatively
Chen, Ma, et al., 2020 [63]	197 Chinese orthopedic patients	Intraoperative total joint arthroplasty	TEG-guided risk stratification	Blood loss, transfusion rate, transfusion volume, DVT	TEG-guided risk stratification for anticoagulation prophylaxis resulted in better safety and equal efficacy as conventional prophylaxis strategy
Qiao and Sun, 2021 [64]	250 Chinese orthopedic patients	Intraoperative	TEG-MA, TEG R-time, TEG-K, TEG alpha-angle	Periprosthetic joint infection (PJI)	TEG parameters predicted PJI
Morda et al., 2017 [65]	32 Italian orthopedic patients	Preoperative, postoperative	TEG-MA, TEG R-time, TEG-K, TEG alpha-angle	Bone cement implantation syndrome (BCIS)	TEG parameters were not predictive of BCIS

ICU

Surgical ICU patients commonly have a myriad of coagulation abnormalities such as thrombocytopenia, prolonged global coagulation times, reduced levels of coagulation inhibitors, or high levels of fibrin split products. Additionally, they are at increased risk of venous thromboembolism due to immobilization, pharmacologic paralysis, repeat surgical procedures, sepsis, mechanical ventilation, vasopressor use, and renal dialysis. Identifying the etiology of these coagulation abnormalities is of utmost importance since each coagulation disorder necessitates different therapeutic strategies. Since TEG provides a comprehensive evaluation of the viscoelastic properties of blood compared to standard plasma assays, in surgical ICU patients TEG has been demonstrated to be predictive of ICU duration, ventilator duration, hospital length of stay, and risk of thromboembolic events [66]. The detection of coagulation abnormalities is even more important in sepsis, a well-known comorbidity during ICU admission since consumption of coagulation factors and subsequent coagulopathy occurs. TEG in this sense has been established to detect coagulopathy and distinguish it among those with and without sepsis so that appropriate management can ensue (Table 6) [66].

**Table 6:** Studies of TEG in ICU perioperative settings

Citation	Patient Sample	Operative Setting	TEG Procedures Assessed	Clinical Outcomes	Summary of Findings
Kashuk et al., 2009 [66]	152 US SICU patients	Intraoperative	r-TEG G	Hypercoagulability, thromboembolic events, transfusion, ICU length of stay, hospital length of stay, ventilator days	TEG-indicated hypercoagulability was predictive of ICU duration, ventilator duration, hospital duration, and risk of thromboembolic event

Cardiovascular

Blood Product Transfusion

The use of TEG in cardiovascular surgeries significantly reduced blood product transfusion compared to clinician-guided practice [67-76]. However, it was not associated with any change in ICU stay or mortality [69, 71, 72]. Redfern et al. 2020 found that TEG-guided protocol significantly reduced blood product use, costs, and reoperation rates; however, it did not impact mortality compared to clinician discretion in 1098 US cardiac patients [74]. Sun et al. 2014 found that TEG-guided protocol was associated with lower fresh frozen plasma (FFP) and platelet transfusion volume without any association with plasma transfusion volume or platelet count in 39 Chinese cardiac patients during ventricular assist device placement [76].

On the other hand, a weak relationship between thromboelastography with platelet mapping (TEG-PM) and platelet transfusion volume was observed in 44 US pediatric cardiac patients [77]. In addition, Westbrook et al. 2009 showed no significant difference in blood product usage between the TEG-guided and the clinician-guided groups in 69 Australian cardiac patients [78].

Bleeding Prediction

The ability of TEG parameters to predict bleeding was questionable in the literature as some studies showed that the use of TEG-MA, TEG R-time, TEG-K, and TEG α-angle was also predictive of blood loss during the operation [75, 79-88] and even postoperatively [89-96]. They could also predict short-term bleeding complications and micro-bleeding [97, 98]. However, they predicted hemostasis only without cyanosis in 63 Italian cardiac patients [99]. Using TEG-MA was useful in predicting long-term ischemic event risk [100], platelet function [101], and “high on-treatment platelet reactivity” [102].

On the other hand, Terada et al. 2019 found that intraoperative use of TEG-MA, TEG R-time, TEG-K, and TEG α-angle was not predictive of blood loss volume in 50 Japanese cardiac patients [103]. Moreover, another five studies showed that these TEG parameters were not predictive of postoperative bleeding [104-108] or even intraoperative bleeding [109, 110].

While other TEG parameters like TEG-PM, rapid thromboelastography maximal amplitude (rTEG-MAf), and rapid thromboelastography fibrinogen level (rTEG-FLEV) were predictive of blood loss volume in cardiac patients [111, 112].

Coagulopathy and Thrombotic Events

Mostly TEG parameters could predict both coagulopathy and thrombotic events. The use of TEG-MA, TEG R-time, TEG-K, and TEG α-angle in cardiac patients was predictive of both coagulopathy [84, 85, 113-120] and even intracranial hemorrhage [120]. Also, they could predict thrombotic events [97, 121] and even pump thrombosis risk [122]. They detected also the P2Y12 inhibition nonresponse, allowing earlier intervention for patients receiving preoperative inhibition therapy in 453 US vascular patients [123]. In comparison to conventional indicators, TEG parameters were better at predicting bleeding and clotting complications [124]. Heparinase modification allowed TEG parameters to diagnose covert coagulopathy [125, 126]. Only Brothers et al. 1993 found that these parameters were not reliably corresponded to clinical coagulopathy in 10 US cardiac patients [127].

Bhardwaj et al. 2017 found that TEG-MA predicted postoperative thrombocytopenia in 35 Indian cardiac patients [128]. In addition, TEG-MA predicted platelet count in cardiac patients [105, 129, 130]. On the other hand, it did not predict adverse events in 233 Danish vascular patients [131].

Other parameters like rTEG-MAf, rTEG-FLEV, TEG-LY60, and TEG-LY150 were also predictive of coagulopathy events in cardiovascular surgeries [132, 133].

Anticoagulant Efficacy Prediction

Intraoperative use of TEG-MA, TEG R-time, TEG-K, and TEG α-angle was effective for monitoring anticoagulant therapy [134-136]. Postoperatively too they were effective for assessing anticoagulation status [137]. TEG-K was found to be effective in monitoring heparin efficacy intraoperatively in 31 US cardiac patients [138]. They also were useful in monitoring anticoagulation reversal in 40 Singaporean vascular patients [106].

TEG-guided intraoperative anticoagulant therapy was effective in 31 US intracranial aneurysm patients [139]; however, when it was compared to traditional methods, no difference was observed in terms of protamine usage or heparin reversal efficacy [140]. TEG-MA was comparable to ROTEM-EXTEM in terms of guiding anticoagulation reversal in 52 UK cardiac patients [141] (Table 7).

**Table 7:** Studies of perioperative TEG in cardiovascular perioperative settings CABG: coronary artery bypass graft; TEG: thromboelastography;  VAD: ventricular assist device; TEG-PM: TEG with platelet mapping; TEG-MA: TEG with maximum amplitude; ROTEM: rotational thromboelastometry; FFP: fresh frozen plasma

Citation	Patient Sample	Operative Setting	TEG Procedures Assessed	Clinical Outcomes	Summary of Findings
Ak et al., 2009 [67]	224 Turkish CABG patient	Intraoperative CABG	TEG-based algorithm vs. clinician-guided practice (RCT)	Volume of blood products transfused	Significantly lower volume of transfusion was required for patients in the TEG-guided condition
Aoki et al., 2012 [68]	100 Japanese vascular surgery patients	Intraoperative	Use of TEG-guided protocol vs. standard of care for transfusion determination (RCT)	Platelet transfusion, bleeding complications	Patients in the TEG-guided protocol group used less platelet transfusion, but had more bleeding complications, compared with the clinician-guided group
Datta and De, 2020 [69]	3000 Indian cardiac patients	Intraoperative	TEG-guided transfusion vs. clinician-guided transfusion policy	Transfusion volume, ICU length of stay, mortality	TEG-guided transfusion resulted in use of lower volume of blood product and no change in ICU stay or mortality
Fleming et al., 2017 [70]	681 US cardiac patients	Intraoperative	Use of TEG-directed transfusion vs. clinician discretion	Blood product usage	Introduction of TEG-directed transfusion procedures reduced volume of blood product used
Hasan et al., 2022 [71]	698 US cardiopulmonary bypass patients	Intraoperative	TEG-guided transfusion protocol vs. conventional testing	Intraoperative blood product usage, postoperative transfusion, mortality	TEG-guided transfusion reduced intraoperative blood product use, but did not reduce postoperative transfusion or mortality rate
Kane et al., 2016 [72]	150 US pediatric cardiac patients	Intraoperative	TEG-guided transfusion vs. clinician-guided transfusion	Blood product usage, postoperative complications	Introduction of TEG-guided transfusion protocol resulted in reduction in blood product usage with no increase in complications
Mendeloff et al., 2009 [73]	112 US neonatal cardiac patients	Intraoperative	TEG-guided transfusion protocol vs. clinician-guided approach	Blood product usage	Introduction of a TEG-guided transfusion protocol resulted in reduced usage of blood product volume
Redfern et al., 2020 [74]	1,098 US cardiac patients	Intraoperative	TEG-guided transfusion protocol vs. clinician discretion	Blood product usage, costs, reoperation rate, mortality	Introduction of TEG-guided transfusion protocol reduced blood product usage, reduced costs, reduced reoperation rates, and did not impact mortality
Shore-Lesserson et al., 1999 [75]	105 US cardiac patients	Intraoperative	TEG-guided transfusion protocol vs. conventional protocol	Blood product usage	TEG-guided transfusion protocol was associated with lower blood product usage volume
Sun et al., 2014 [76]	39 Chinese cardiac patients	Intraoperative VAD placement	TEG-directed transfusion protocol vs. clinician discretion	Coagulopathy, platelet count, blood product usage	TEG-directed transfusion was associated with lower FFP and platelet transfusion volume, but there was no difference in plasma transfusion volume or platelet count
Barker et al., 2019 [77]	44 US pediatric cardiac patients	Intraoperative and postoperative	TEG-PM	Mortality, platelet transfusion volume	Weak relationship between TEG-PM and platelet transfusion volume
Westbrook et al., 2009 [78]	69 Australian cardiac patients	Intraoperative	TEG-guided transfusion protocol vs. clinician discretion (RCT)	Blood product usage	There was a no significant difference in blood product usage between the TEG-guided and clinician discretion groups
Emani et al., 2021 [79]	703 US pediatric cardiac patients	Intraoperative	TEG-MA	Perioperative bleeding, transfusion volume	TEG-MA was predictive of bleeding volume. TEG-MA guidance had utility for reducing transfusion volume
Emani et al., 2018 [80]	511 US pediatric cardiac patients	Intraoperative	TEG-MA	Intraoperative bleeding	TEG-MA predicts intraoperative bleeding volume
Essell et al., 1993 [81]	36 US cardiopulmonary bypass patients	Intraoperative	TEG-MA, TEG R-time, TEG-K, TEG alpha-angle	Intraoperative bleeding	TEG parameters were predictive of hemorrhage risk
Sivapalan et al., 2017 [82]	199 Danish cardiac patients	Intraoperative CABG	TEG-MA	Transfusion volume	TEG-MA was predictive of transfusion volume
Liu et al., 2021 [83]	398 Chinese vascular patients	Preoperative, intraoperative	TEG-MA, TEG R-time, TEG-K, TEG alpha-angle	Blood loss, hemorrhage, transfusion	TEG parameters were predictive of blood loss volume, hemorrhage, and transfusion
Sharma et al., 2018 [84]	50 Indian cardiac patients	Intraoperative	TEG-MA, TEG R-time, TEG-K, TEG alpha-angle	Coagulopathy, blood loss volume	TEG parameters predicted coagulopathy and blood loss volume
Tuman et al., 1989 [85]	42 US cardiac patients	Intraoperative cardiopulmonary bypass	TEG-MA, TEG R-time, TEG-K, TEG alpha-angle	Bleeding, hemorrhage, coagulopathy	TEG parameters were predictive of bleeding volume, hemorrhage, and coagulopathy
Moganasundram et al., 2010 [86]	50 UK pediatric cardiac patients	Intraoperative	TEG-MA, TEG R-time, TEG-K, TEG alpha-angle	Bleeding	TEG parameters predicted bleeding
Nuttall et al., 1997 [87]	82 US cardiac patients	Preoperative, intraoperative cardiopulmonary bypass	TEG-MA, TEG R-time, TEG-K, TEG alpha-angle	Intraoperative bleeding	TEG parameters predicted subjective clinician judgment of excessive bleeding
Singh et al., 2015 [88]	55 Indian cardiac patients	Intraoperative coronary bypass	TEG-MA	Blood loss	TEG-MA was predictive of blood loss volume
Cammerer et al., 2003 [89]	255 German cardiac patients	Preoperative, intraoperative	TEG alpha-angle	Platelet function, surgical bleeding, postoperative bleeding	TEG alpha-angle is a strong predictor of postoperative bleeding
Martin et. al., 1991 [90]	22 UK pediatric cardiac patients	Preoperative, postoperative	TEG-MA, TEG R-time, TEG-K, TEG alpha-angle	Postoperative bleeding	TEG parameters predicted postoperative bleeding
Muller et al., 1975 [91]	9 German cardiac patients	Intraoperative	TEG-MA, TEG R-time, TEG-K, TEG alpha-angle	Postoperative bleeding	TEG parameters predicted postoperative bleeding
Niebler et al., 2012 [92]	60 US cardiac patients	Preoperative, intraoperative cardiopulmonary bypass	TEG-MA, TEG R-time, TEG-K, TEG alpha-angle	Postoperative bleeding	TEG parameters are predictive of postoperative bleeding
Preisman et al., 2010 [93]	59 Israeli vascular patients	Preoperative	TEG-MA, TEG R-time, TEG-K, TEG alpha-angle	Excessive blood loss	TEG parameters predicted excessive postoperative blood loss
Shih et al., 1997 [94]	43 Chinese cardiac patients	Intraoperative cardiopulmonary bypass	TEG-MA, TEG R-time, TEG-K, TEG alpha-angle	Postoperative bleeding	TEG parameters are predictive of postoperative bleeding
Smith et al., 2020 [95]	120 US cardiac patients	Intraoperative cardiopulmonary bypass	TEG-MA, TEG R-time, TEG-K, TEG alpha-angle	Postoperative bleeding	TEG parameters are associated with postoperative bleeding
Williams et al., 1999 [96]	494 US pediatric cardiac patients	Intraoperative cardiopulmonary bypass	TEG-MA, TEG R-time, TEG-K, TEG alpha-angle	Postoperative bleeding	TEG parameters predicted postoperative bleeding
Rymuza et al., 2018 [97]	54 Polish vascular patients	Preoperative, intraoperative TAVI	TEG-MA, TEG R-time, TEG-K, TEG alpha-angle	Bleeding complications	TEG parameters predicted short-term bleeding complications
Xu et al., 2016 [98]	261 Chinese vascular patients	Intraoperative SAC embolization	TEG-MA, TEG R-time, TEG-K, TEG alpha-angle	Microbleeding complications	TEG parameters predicted microbleeding
Rizza et al., 2017 [99]	63 Italian cardiac patients	Intraoperative cardiopulmonary bypass	TEG-MA, TEG R-time, TEG-K, TEG alpha-angle	Cyanosis, hemostasis	TEG parameters were predictive of hemostasis but not cyanosis
Hou et al., 2017 [100]	759 Chinese vascular patients	Intraoperative PCI	TEG-MA, TEG R-time, TEG-K, TEG alpha-angle	Platelet function, long-term ischemic events	TEG-MA predicted long-term ischemic event risk
Kirmani et al., 2017 [101]	50 UK cardiac patients	Preoperative	TEG-MA	Platelet function	TEG-MA is predictive of platelet function
Cheng et al., 2020 [102]	110 Chinese vascular patients	Intraoperative PCI	TEG R-time, TEG-MA	Identification of high-on treatment platelet reactivity (HTPR)	TEG parameters were effective for predicting HTPR complications
Terada et al., 2019 [103]	50 Japanese cardiac patients	Intraoperative	TEG-MA, TEG R-time, TEG-K, TEG alpha-angle	Blood loss	TEG parameters were not predictive of blood loss volume
Carroll et al., 2006 [104]	19 US cardiac patients	Intraoperative	TEG-MA, TEG R-time, TEG-K, TEG alpha-angle	Postoperative bleeding	TEG parameters were not related to postoperative bleeding
Pekelharing et al., 2013 [105]	107 UK pediatric cardiac patients	Postoperative cardiopulmonary bypass	TEG-MA, TEG R-time, TEG-K, TEG alpha-angle	Postoperative bleeding, platelet count	TEG-MA is associated with platelet count, but TEG parameters are not predictive of postoperative bleeding
Ti et al., 2002 [106]	40 Singaporean vascular patients	Preoperative CABG	TEG-MA, TEG R-time, TEG-K, TEG alpha-angle	Anticoagulation reversal monitoring, bleeding	TEG parameters were not predictive of postoperative bleeding but were useful for monitoring anticoagulation reversal
Welsh et al., 2014 [107]	76 US cardiac patients	Intraoperative cardiopulmonary bypass	TEG-MA, TEG R-time, TEG-K, TEG alpha-angle	Postoperative bleeding, cause of bleeding	TEG parameters did not predict postoperative blood loss, and did not distinguish causes of bleeding
Agarwal et al., 2006 [108]	54 UK cardiac patients	Coronary artery bypass surgery, preoperative and postoperative	TEG-PM post-operative	Post-bypass platelet function; blood loss at 4 hours; blood loss at 12 hours	TEG-PM post-operative was not related to any outcomes; authors recommend using pre-operative measures to predict outcomes
Dorman et al., 1993 [109]	60 US vascular patients	Intraoperative	TEG-MA, TEG R-time, TEG-K, TEG alpha-angle	Blood loss	TEG parameters did not predict blood loss
Sharma et al., 2014 [110]	439 US cardiac patients	Intraoperative	TEG-MA, TEG alpha-angle	Bleeding volume	TEG parameters did not improve prediction of bleeding volume
Weitzel et al., 2012 [111]	40 US cardiac patients	Intraoperative	TEG-PM	Postoperative blood loss	TEG parameters predicted postoperative blood loss volume
Miao et al., 2014 [112]	100 Chinese pediatric cardiac patients	Intraoperative	rTEG-MAf, rTEG-FLEV	Blood loss, transfusion volume	rTEG parameters were related to postoperative blood loss volume
Koster et al., 2001 [113]	19 German cardiopulmonary bypass patients	Intraoperative	TEG-MA, TEG R-time, TEG-K, TEG alpha-angle	Coagulopathy	TEG parameters predicted coagulopathy and were useful in guiding intraoperative treatment
Miller et al., 2000 [114]	85 US pediatric cardiac patients	Intraoperative	TEG-MA, TEG R-time, TEG-K, TEG alpha-angle	Coagulopathy	TEG parameters predicted coagulopathy
Spiess et al., 1987 [115]	38 US cardiac patients	Preoperative, intraoperative	TEG-MA, TEG R-time, TEG-K, TEG alpha-angle	Coagulopathy	TEG parameters predicted coagulopathy
Vlot et al., 2021 [116]	89 Dutch cardiac patients	Intraoperative	TEG-MA, TEG R-time, TEG-K, TEG alpha-angle	Coagulopathy	TEG parameters were associated with coagulopathy
Yamamoto et al., 2021 [117]	40 Japanese cardiac patients	Intraoperative	TEG-MA	Coagulopathy	TEG-MA was predictive of coagulopathy
Yan et al., 2021 [118]	521 Chinese cardiac patients	Intraoperative	TEG-MA	Hypercoagulability	TEG-MA values predicted hypercoagulable states
Zisman et al., 2009 [119]	62 Israeli cardiac patients	Intraoperative	TEG-MA, TEG R-time, TEG-K, TEG alpha-angle	Coagulopathy	TEG parameters predicted postoperative coagulopathy
Liang et al., 2020 [120]	240 Chinese ischemic stroke patients	Preoperative	TEG-MA, TEG R-time, TEG-K, TEG alpha-angle	Coagulopathy, intracranial hemorrhage	TEG parameters were predictive of coagulopathy and intracranial hemorrhage
Tuman et al., 1991 [121]	80 US vascular patients	Intraoperative	TEG-MA, TEG R-time, TEG-K, TEG alpha-angle	Thrombotic events, coagulopathy	TEG parameters predicted thrombotic events
Xia et al., 2020 [122]	90 US cardiac patients	Intraoperative, postoperative LVAD placement	TEG-MA, TEG R-time, TEG-K, TEG alpha-angle	Pump thrombosis	Differences in the rate of change in TEG parameters over time in the postoperative period predicted risk of pump thrombosis
Rogers et al., 2021 [123]	453 US vascular patients	Intraoperative CABG	TEG-MA, TEG R-time, TEG-K, TEG alpha-angle	Detection of P2Y12 inhibition	TEG parameters detected P2Y12 inhibition nonresponse, allowing earlier intervention for patients receiving preoperative inhibition therapy
Mack et al., 2021 [124]	25 US vascular patients	Preoperative, intraoperative	TEG-MA, TEG R-time, TEG-K, TEG alpha-angle	Bleeding, platelet count, clotting complications	TEG parameters provided more accurate indication of bleeding and clotting complications compared with conventional indicators
Tuman et al., 1994 [125]	51 US cardiac patients	Intraoperative	TEG-MA, TEG R-time, TEG-K, TEG alpha-angle	Anticoagulation monitoring	Heparinase modification can be combined with TEG parameters to enable monitoring of coagulation status in the presence of anticoagulants
Yabrodi et al., 2022 [126]	100 US pediatric cardiac ECMO patients	Intraoperative	TEG-MA, TEG R-time, TEG-K, TEG alpha-angle	Coagulopathy	Heparinase modification may allow TEG parameters to diagnose covert coagulopathy
Brothers et al., 1993 [127]	10 US cardiac patients	Intraoperative abdominal aortic aneurysm surgery	TEG-MA, TEG R-time, TEG-K, TEG alpha-angle	Coagulopathy	TEG parameters did not reliably correspond to clinical coagulopathy. Authors suggest the clinical value of TEG is not supported
Bhardwaj et al., 2017 [128]	35 Indian cardiac patients	Intraoperative cardiac bypass	TEG-MA, ROTEM-FIBTEM	Coagulopathy, chest drain output	TEG-MA predicts postoperative thrombocytopenia, ROTEM-FIBTEM predicts postoperative hyperfibrinogenemia
Bhatia et al., 2017 [129]	4 German pediatric cardiac patients	Postoperative VAD placement	TEG-MA, TEG R-time, TEG-K, TEG alpha-angle	Platelet count	TEG-MA is a strong predictor of platelet count. Authors recommend using r-TEG in preference to traditional laboratory measures
Gautam et al., 2017 [130]	105 US pediatric cardiac patients	Intraoperative	FFTEG	Platelet count	FFTEG values are predictive of platelet count
Dridi et al., 2014 [131]	233 Danish vascular patients	Intraoperative percutaneous coronary intervention (PCI)	TEG-MA	Adverse events	TEG-MA did not predict adverse events
Miao et al., 2015 [132]	80 Chinese pediatric cardiac patients	Intraoperative	rTEG-MAf, rTEG-FLEV	Coagulopathy	rTEG parameters were predictive of coagulopathy
Vanek et al., 2007 [133]	65 Czech vascular patients	Postoperative	TEG-LY60, TEG-LY150	Coagulopathy	TEG parameters were associated with coagulopathy
Koster et al., 2008 [134]	15 German cardiopulmonary bypass patients	Intraoperative	TEG-MA, TEG R-time, TEG-K, TEG alpha-angle	Bivalirudin anticoagulation	TEG parameters are effective for intraoperative monitoring of anticoagulation therapy
Martin et al., 1994 [135]	15 UK vascular patients	Preoperative, intraoperative	TEG-MA, TEG R-time, TEG-K, TEG alpha-angle	Anticoagulation monitoring	TEG parameters are more useful for intraoperative anticoagulation monitoring than conventional tests
Wasowicz et al., 2009 [136]	38 Canadian cardiac patients	Intraoperative	TEG-MA, TEG R-time, TEG-K, TEG alpha-angle	Anticoagulation monitoring	TEG parameters were more effective than conventional laboratory measures at monitoring intraoperative anticoagulation
Murray et al., 1997 [137]	36 US vascular patients	Intraoperative	TEG-MA, TEG R-time, TEG-K, TEG alpha-angle	Anticoagulation detection	TEG was effective at assessing postoperative anticoagulation status
Chavez et al., 2004 [138]	31 US cardiac patients	Intraoperative cardiopulmonary bypass	TEG TF/K	Heparin anticoagulation efficacy	TEG parameters were effective for monitoring heparin efficacy intraoperatively
McTaggart et al., 2015 [139]	31 US intracranial aneurysm patients	Intraoperative	TEG-guided anticoagulant therapy	Platelet function, complications	TEG-guided intraoperative anticoagulant therapy was effective
Levin et al., 2014 [140]	82 South African coronary bypass patients	Intraoperative	Use of TEG-guided anticoagulation compared with conventional methods	Protamine dosage, heparin reversal	TEG-guided anticoagulation methods did not differ from traditional methods in terms of protamine usage or heparin reversal efficacy
Ortmann et al., 2015 [141]	52 UK cardiac patients	Intraoperative	TEG-MA, ROTEM-EXTEM	Anticoagulation reversal detection	TEG and ROTEM parameters were comparable in terms of guiding anticoagulation reversal

Transplant

Perioperative TEG is used in organ transplantation surgeries such as liver, kidney, pancreas-kidney, or bowel because of their abilities in the prediction of coagulopathy and thrombotic events. While Abuelkasem et al. found that TEG-R could not predict coagulopathy in liver transplant surgeries as effectively as ROTEM [142], other studies have demonstrated that TEG parameters like TEG-MA, TEG R-Time, TEG-K, and TEG α-angle could predict or be related to coagulopathy [143-149]. Despite the relation of TEG parameters to coagulopathy, they were not related to bleeding time [144] which was supported by Sujka et al. who compared TEG-directed transfusion protocol and the clinician-directed transfusion system and found no difference between both methods in decreasing the blood loss amount [150].

Also, TEG parameters predicted hypercoagulable status and thrombotic events [149, 151-153] even in comparison to the conditional laboratory tests [154] while Krzanicki et al. found that they could predict hypercoagulable status only without thrombotic events in liver transplant patients [155]. On the other hand, Sujka et al. found that TEG-directed blood transfusion increased the thromboembolic events compared to the clinician-directed protocol in liver transplant patients [150].

Regarding the use of blood products, the studies revealed different results. TEG parameters reduced the usage of blood products [156-158]; however, in comparison to other conventional tests or clinician-directed transfusion system, no differences were observed except for Sujka et al. who found TEG-directed transfusion system reduced only FFP use between other blood products [150, 159, 160]. Coakley et al. investigated both TEG and ROTEM parameters and found that ROTEM improved clinicians’ decisions compared to TEG usage [161].

In postoperative outcomes like survival, graft function, and hospital stay, controversial results were observed in the studies. Sam et al. found that TEG did not relate to renal graft function while Walker et al. found that it is an indicator of graft function [146, 162]. This controversy was seen also in the prediction of liver cirrhosis [148, 163]. TEG’s usage was not associated with mortality or survival rates [156, 160]. On the other hand, it decreased hospital stay length and reoperation needs [147, 160] (Table 8).

**Table 8:** Studies of TEG in transplant perioperative settings TEG: thromboelastography; TEG-PM: TEG with platelet mapping; TEG-MA: TEG with maximum amplitude; ROTEM: rotational thromboelastometry; TEG-CI: TEG with coagulation index; FFP: fresh frozen plasma

Citation	Patient Sample	Operative Setting	TEG Procedures Assessed	Clinical Outcomes	Summary of Findings
Abuelkasem et al., 2016 [142]	36 US liver transplant patients	Intraoperative liver transplant	TEG R-time; ROTEM CT (INTEM-CT and EXTEM-CT)	Coagulopathy	INTEM-CT and EXTEM-CT were effective predictors of coagulopathy, but TEG-R was not
Burke et al., 2004 [143]	85 US diabetic simultaneous pancreas-kidney (SPK) transplant patients and 54 non-diabetic kidney transplant patients	Intraoperative during SPK or kidney transplant surgery	TEG-MA, TEG R-time, TEG-K, TEG alpha-angle	Coagulopathy	TEG parameters were useful for guiding transplant surgery and were validated in SPK for diabetic patients
Davis & Chandler, 1995 [144]	120 US kidney transplant patients	Intraoperative renal biopsy	TEG-K, TEG alpha-angle	Bleeding time, coagulopathy	TEG parameters were related to coagulopathy but not to bleeding time
Kettner et al., 1998 [145]	72 Austrian liver transplant patients	Intraoperative	TEG-MA, TEG R-time, TEG-K, TEG alpha-angle	Coagulopathy	TEG parameters can distinguish between some causes of bleeding complications
Sam et al., 2021 [146]	25 Indian kidney transplant patients	Intraoperative	TEG R-time, TEG-CI, TEG-MA	Coagulopathy, graft function	TEG parameters were related to coagulopathy but not graft function
Schulick et al., 2020 [147]	40 US liver transplant patients	Intraoperative	TEG-MA, TEG R-time, TEG-K, TEG alpha-angle	Coagulopathy, re-operation, length of stay	TEG parameters were predictive of coagulopathy, re-operation, and length of hospital stay
Tanner et al., 2018 [148]	33 US liver transplant patients	Intraoperative	TEG-MA, TEG R-time, TEG-K, TEG alpha-angle	Coagulopathy, cirrhosis	TEG parameters predict coagulopathy but do not differentiate between patients with and without postoperative cirrhosis
Raveh et al., 2018 [149]	48 US visceral transplant patients	Intraoperative	TEG-MA, TEG R-time, TEG-K, TEG alpha-angle	Thrombotic complications, hemorrhagic complications	TEG parameters were predictive of thrombotic and hemorrhagic complications
Sujka et al., 2018 [150]	38 US pediatric liver transplant patients	Intraoperative	TEG-directed transfusion protocol vs. clinician discretion	Blood loss, blood product usage, FFP use, thromboembolic complications	Introduction of TEG-directed transfusion protocol was associated with decreased FFP usage, but no overall change in blood product usage or blood loss. Thromboembolic complications increased
De Pietri et al., 2020 [151]	27 Italian liver transplant patients	Intraoperative and postoperative	TEG-MA, TEG R-time, TEG-K, TEG alpha-angle, TEG-G, TEG-LY30, TEG-LY60	Portal vein thrombosis (PVT), hepatic artery thrombosis (HAT)	TEG-G and TEG-LY60 were predictive of PVT and HAT events
Eldeen et al., 2016 [152]	828 UK liver transplant patients	Preoperative	TEG-MA, TEG R-time, TEG-K, TEG alpha-angle	Early hepatic artery thrombosis (E-HAT)	TEG parameters predict E-HAT
Pivalizza et al., 1998 [153]	19 Italian bowel transplant patients	Intraoperative	TEG-MA, TEG R-time, TEG-K, TEG alpha-angle, TEG-CL50	Hypocoagulation	TEG-MA was related to hypocoagulation
Garg et al., 2021 [154]	50 Indian kidney donors	Preoperative, postoperative	TEG-MA, TEG R-time, TEG-K, TEG alpha-angle	Postoperative hypercoagulability	TEG parameters were better predictors of postoperative hypercoagulability compared with traditional lab tests
Krzaniki et al., 2013 [155]	124 UK liver transplant patients	Intraoperative	TEG-MA, TEG R-time, TEG-K, TEG alpha-angle	Hypercoagulability, thrombotic complications	TEG parameters were predictive of hypercoagulability but not thrombotic complications
De Pietri et al., 2015 [156]	386 Italian liver transplant patients	Intraoperative	Comparison of two alternate TEG-based transfusion algorithms	Blood product volume, mortality	Improved algorithm employing additional TEG measures of functional fibrinogen and maximum amplitude of functional fibrinogen resulted in reduced blood product usage with no change in mortality
Kang et al., 1985 [157]	66 US liver transplant patients	Intraoperative	TEG-guided transfusion	Blood product usage	TEG-guided transfusion protocol was associated with reduction in blood product usage
Zamper et al., 2018 [158]	237 Brazilian liver transplant patients	Intraoperative liver transplant	TEG-guided transfusion protocol vs. clinician discretion	Blood product usage	Introduction of TEG-guided transfusion protocol reduced blood product usage volume
Gaspari et al., 2021 [159]	226 Italian liver transplant patients	Intraoperative	TEG-guided vs. conventional coagulation test (CCT)-guided transfusion strategies	Blood product usage	After propensity matching, there was no difference between blood product usage between TEG and CCT-guided transfusion techniques
Gopal et al., 2020 [160]	68 UK pancreas-kidney transplant patients	Intraoperative	TEG-directed vs. conventional anticoagulation protocol	Blood product usage, hospital length of stay, 1 year survival	The TEG-directed anticoagulation protocol resulted in reduced blood product usage and shorter length of stay, with no difference in survival
Coakley et al., 2006 [161]	20 UK liver transplant patients	Intraoperative	Kaolin TEG, kaolin heparinase TEG, ROTEM-NITEM, ROTEM-FIBTEM	Time to administer blood transfusion	TEG and ROTEM parameters differed on transfusion guidance, with ROTEM judged to have made better clinical decisions
Walker et al., 2020 [162]	71 US kidney transplant patients	Preoperative, postoperative	TEG-LY30	Graft function	TEG-LY30 was predictive of good graft function
Kohli et al., 2019 [163]	164 US liver transplant patients	Preoperative	TEG-MA, TEG R-time, TEG-K, TEG alpha-angle	Cirrhosis severity	TEG parameters identified cirrhosis severity

Miscellaneous 

TEG is used in many other sites involving neurological, gastrointestinal, general, cardiopulmonary, plastic, urological, and oncological procedures.

Neurological

TEG parameters showed an evitable role in improving hematological outcomes in people who underwent neurosurgeries whether they were adults [164-168] or children [169]. TEG parameters like TEG-R, TEG-MA, TEG-K, and TEG α-angle could predict hypercoagulation or thrombotic complications [164, 166, 168, 169]; however, compared to control treatment, no difference was observed [170]. In addition, these parameters predicted bleeding and hypo-coagulation status whether intraoperative or postoperative [166, 169, 171, 172] besides using them could decrease bleeding complications risk compared to other conventional labs [170]. TEG-guided transfusion was effective to decrease the transfusion of blood products compared to the clinician-guided protocol [172]. Also, TEG-guided use of intraoperative antiplatelet therapy succeeded to prevent major complications [167] while only TEG-R was rare to be associated with postoperative complications [164].

Only TEG-PM could not predict thrombotic events or even bleeding complications through neurosurgical procedures [173]; however, it showed good ability in the prediction of platelet inhibition in comparison to other modalities [165]. 

Gastrointestinal

TEG parameters showed promising results in gastroenterology surgeries [174]. Using TEG in bariatric surgeries could predict hypercoagulability conditions [175-177] and this ability especially increased in females and older patients [175]. However, in liver-related surgeries, TEG efficacy was controversial as Oo 2020 et al. and Vieira da Rocha 2009 et al. showed that the essential TEG parameters were not predictive of ulcerative bleeding risk or hemostasis variation [178, 179]. On the other hand, Okida 1991 et al. and Zanetto 2021 et al. showed the efficacy of these parameters in the prediction of coagulopathy and perioperative bleeding [180, 181]. Moreover, compared to clinician-guided transfusion, TEG-guided transfusion decreased the usage of blood products; however, it was not different to reduce the complications rate [182]. TEG usage could not predict postoperative sepsis in oesophagectomy surgeries [183]. In patients with obstructive jaundice, TEG parameters also could not predict coagulopathy or platelet function during their surgeries for drainage of obstructive jaundice [184] while they predicted bleeding and coagulopathy in cystectomy operations [185, 186]. Also, they could predict deep venous thrombosis risk in gastric cancer patients comorbid with portal hypertension [187].

General 

Few studies investigated the role of TEG among pediatric patients undergoing general surgical procedures and they found that applying TEG or ROTEM in pediatric patients increased coagulopathy risk and blood products use [188] while in neonates, TEG parameters predicted sepsis early [189]. Also, TEG-guided transfusion decreased blood products use compared to clinician-guided transfusion while in mortality and morbidity risks, no differences were detected [190]. The use of TEG among adults undergoing general surgical procedures was better described in the literature. They were effective in the prediction of bleeding [191]. Using TEG-PM in monitoring platelet inhibition in patients on clopidogrel was useful in decreasing unneeded treatment cancellations besides the patient risk [192]. However, comparing the conventional transfusion protocol to TEG-guided transfusion revealed no significant difference in detecting bleeding [193]. The conventional TEG parameters with the celite-activated ones were predictive or associated with hypercoagulability or thrombotic events [194-196]. Coagulopathy prediction was achieved also by TEG-guided transfusion compared to the use of conventional methods [193]. Also, they showed better prediction values of survival rates compared to other conventional methods [197]. On the other hand, TEG-guided transfusion was not different to the conventional protocol in the prediction of mortality [193]. They could predict the blood products use [191] and using TEG-guided transfusion was effective in reducing the need for blood products [193]. Moreover, they resembled a good option to guide the optimal treatment, especially in patients comorbid with Gaucher disease who undergoing general surgeries [198]. In flap operations, the TEG parameters could not predict the flap loss risks [199]; however, they were predictive of coagulopathy and thrombotic events [200]. Also, in maxillary surgeries, they could predict both bleeding and platelet count [201]. 

Extracorporeal Membrane Oxygenation (ECMO)

Applying TEG in surgical procedures in patients on ECMO was controversial in the literature in both adults and pediatric patients. In pediatric patients, TEG-guided anticoagulation protocol significantly reduced blood products usage, decreased complications, and increased ECMO circuit life compared to the clinician-guided system [202, 203] which was supported by Moynihan 2017 et al. who found that they were useful in monitoring intraoperative anticoagulation [204]. Moreover, TEG-R significantly predicted thrombotic events [205]. On the other hand, the bleeding complications predictive value of conventional TEG parameters was controversial as Saini et al. showed that they could not predict bleeding [206] while Sleeper et al. found that these parameters predicted bleeding [207]. Also, TEG kaolin and heparinase had a poor indication ability of aPTT and an acceptable indication of platelet count which recommended the usage of conventional laboratory tests [208]. Regarding their use in adult patients on ECMO, TEG-R, ROTEM-INTEM, and conventional methods had the same efficacy in anticoagulation monitoring [209]. Also, the TEG flat line reading had no relation to the perioperative bleeding [210]. However, other studies showed that the conventional TEG parameters were effective to monitor anticoagulation [211] and to predict coagulopathy in adults on ECMO patients [212]. 

Others

TEG was also used in monitoring hematological outcomes in urological procedures such as prostatectomy [213, 214] and renal biopsy [215] or even in nephrotic syndrome patients [216]. However, its efficacy was questionable as in prostatectomy procedures, TEG clot lysis correlated with bleeding [214] while other parameters like TEG-LY30 and TEG-LY40 were not able to predict postoperative coagulopathy [213]. Also, during the renal biopsy, TEG-MA was not effective to predict bleeding time [215]. However, TEG parameters like TEG-MA, TEG-R, TEG-K, and TEG α-angle were associated with coagulopathy complications and could distinguish different renal pathologies in 713 Chinese nephrotic syndrome patients [216]. TEG parameters were predictive in oncology patients regarding platelet count, hypercoagulability, tumor type, resection success, and postoperative complications [217-219]. Also, they were useful in monitoring the anticoagulation status in patients who underwent thoracic surgeries [220] and patients on mechanical circulatory support devices [221]** **(Table 9).

**Table 9:** Studies of TEG in miscellaneous perioperative settings TEG: thromboelastography; TEG-PM: TEG with platelet mapping; TEG-MA: TEG with maximum amplitude; ROTEM: rotational thromboelastometry; aPTT: activated partial thromboplastin time

Citation	Patient Sample	Operative Setting	TEG Procedures Assessed	Clinical Outcomes	Summary of Findings
Abrahams et al., 2002 [164]	46 US neurosurgery patients	Intraoperative craniotomy	TEG R-time	Hypercoagulability, DVT, hematoma	TEG was useful for predicting hypercoagulability throughout procedure; post-operative adverse outcomes were too rare to be statistically associated with TEG parameters
Corliss et al., 2017 [165]	23 US neurosurgery patients	Intraoperative	TEG-PM	Platelet inhibition	TEG-PM provides a better indicator of platelet inhibition compared with other methodologies
Javed et al., 2021 [166]	118 US intracranial aneurysm patients	Intraoperative	TEG-MA, TEG R-time, TEG-K, TEG alpha-angle	Complications	TEG parameters were predictive of hemorrhagic and ischemic complications
Wu et al., 2019 [167]	183 Chinese neurosurgery patients	Intraoperative cerebrovascular stent placement	TEG-guided intraoperative antiplatelet therapy	Major complications	TEG-guided therapy was effective at avoiding major complications in the context of intraoperative antiplatelet therapy
Parker et al., 2012 [168]	39 UK head and neck surgery patients	Preoperative free tissue transfer	TEG-MA, TEG R-time, TEG-K, TEG alpha-angle	Thrombotic complications	TEG parameters are predictive of thrombotic complications
El Kady et al., 2009 [169]	40 Egyptian pediatric neurosurgery patients	Preoperative, intraoperative, postoperative	TEG-MA, TEG R-time, TEG-K, TEG alpha-angle	Hypocoagulation	TEG parameters predicted hypocoagulation
Li et al., 2021 [170]	188 Chinese cranial patients	Intraoperative	TEG-guided treatment vs. control	Bleeding, complications	TEG-guided treatment resulted in less bleeding and no difference in thrombotic complications
Zhang et al., 2017 [171]	181 Chinese neurosurgery patients	Intraoperative	TEG-MA, TEG R-time, TEG-K, TEG alpha-angle	Intraoperative blood loss	TEG parameters predicted intraoperative blood loss
Zhou et al., 2019 [172]	82 Chinese intracerebral hemorrhage patients	Intraoperative	TEG-guided transfusion protocol vs. clinician discretion	Transfusion volume, bleeding outcomes	TEG-guided transfusion resulted in reduced intraoperative and postoperative bleeding and in lower transfusion volumes
Corliss et al., 2020 [173]	191 US neurosurgery patients	Intraoperative	TEG-PM	Hemorrhagic complications, thrombotic complications	TEG-PM parameters are not predictive of hemorrhagic or thrombotic complications
Mahla et al., 2001 [174]	20 Austrian abdominal surgery patients	Preoperative, postoperative	TEG-MA, TEG R-time, TEG-K, TEG alpha-angle	Postoperative hypercoagulability	TEG parameters detect postoperative hypercoagulability up to a week after surgery that conventional diagnostics do not detect
Duman Guven et al., 2020 [175]	54 Turkish bariatric patients	Preoperative, postoperative	TEG-MA	Hypercoagulability	TEG-MA is predictive of hypercoagulability in morbidly obese patients, and is more predictive among female patients and older patients
Kupcinskiene et al., 2017 [176]	60 Lithuanian bariatric patients	Preoperative, postoperative	TEG-MA, TEG R-time, TEG-K, TEG alpha-angle	Hypercoagulability	TEG parameters were useful for perioperative monitoring of coagulability
Cowling et al., 2021 [177]	422 US bariatric surgery patients	Preoperative	TEG-MA, TEG R-time, TEG-K, TEG alpha-angle	Coagulopathy	Preoperative TEG parameters predict postoperative coagulopathy in morbidly obese patients
Oo et al., 2020 [178]	41 Australian liver surgery patients	Intraoperative	TEG-MA, TEG R-time, TEG-K, TEG alpha-angle	Hemostasis	TEG parameters did not accurately indicate variations from hemostasis
de Rocha et al., 2009 [179]	150 Brazilian hepatic patients	Intraoperative variceal band ligation	TEG-MA, TEG R-time, TEG-K, TEG alpha-angle	Ulcerative bleeding	TEG parameters were unrelated to risk of ulcerative bleeding
Okida et al., 1991 [180]	16 Japanese liver surgery patients	Preoperative, intraoperative	TEG-MA, TEG R-time, TEG-K, TEG alpha-angle	Coagulopathy	TEG parameters predict coagulopathy
Zanetto et al., 2021 [181]	80 US cirrhosis patients	Intraoperative	TEG-MA, TEG R-time, TEG-K, TEG alpha-angle	Perioperative bleeding	TEG parameters predicted perioperative bleeding
Vuyyuru et al., 2020 [182]	58 Indian liver disease patients	Intraoperative	TEG-guided transfusion protocol vs. clinician discretion (RCT)	Blood product usage, complications	The TEG-guided transfusion protocol resulted in lower blood product usage volume with no difference in complications
Durila et al., 2012 [183]	43 Czech oesophagectomy patients	Preoperative, postoperative	TEG-MA, TEG R-time, TEG-K, TEG alpha-angle	Postoperative sepsis	TEG parameters did not predict sepsis
Cakir et al., 2009 [184]	23 obstructive jaundice Turkish patients	Intraoperative during surgery for drainage of obstructive jaundice	TEG-MA, TEG R-time, TEG-K, TEG alpha-angle	Coagulopathy, platelet function	No effects detected
Rasmussen et al., 2015 [185]	40 Danish cystectomy patients	Intraoperative	TEG-MA, TEG R-time, TEG-K, TEG alpha-angle	Hemorrhage, coagulation competence	TEG parameters were predictive of blood loss and coagulopathy
Rasmussen et al., 2016 [186]	39 Danish cystectomy patients	Intraoperative	TEG-MA	Blood loss	TEG-MA was related to blood loss volume
Gong et al., 2021 [187]	172 Chinese gastric cancer patients with portal hypertension	Preoperative, postoperative	TEG-MA, TEG R-time, TEG-K, TEG alpha-angle	Occurrence of DVT	TEG parameters were predictive of DVT risk
Burton et al., 2021 [188]	265 US pediatric general surgery patients	May vary (retrospective registry study)	TEG or ROTEM vs. no use of viscoelastic testing	Coagulopathy, blood product use	Patients receiving TEG or ROTEM had more coagulopathy and used more blood products than other patients
Grant & Hadley, 1997 [189]	103 South African neonatal general surgery patients	Postoperative	TEG-MA, TEG R-time, TEG-K, TEG alpha-angle	Differentiation of patients with and without sepsis	TEG parameters were effective at early identification of neonatal sepsis
Raffaeli et al., 2022 [190]	139 Italian neonatal general surgery patients	Intraoperative	TEG-guided transfusion protocol vs. clinician discretion	Blood product usage, mortality, morbidity	Introduction of a TEG-guided transfusion protocol decreased blood product usage volume and did not impact mortality or morbidity
Zhang et al., 2014 [191]	55 Chinese general surgery patients	Intraoperative	TEG-MA, TEG R-time, TEG-K, TEG alpha-angle	Postoperative bleeding, blood product usage	TEG parameters predicted postoperative bleeding and blood product usage
Kasivisvanathan et al., 2014 [192]	182 UK general surgery patients taking clopidogrel therapy	Intraoperative	Stratification of bleeding risk by TEG-PM	Detection of platelet inhibition	TEG-PM was effective at minimizing patient risk
Shi et al., 2019 [193]	74 Chinese general surgery patients	Intraoperative	TEG-guided transfusion protocol vs. conventional protocol	Coagulopathy, blood product usage, blood loss, bleeding, mortality	TEG-guided transfusion reduced blood product usage, and TEG estimated coagulopathy better, but there was no difference between groups in bleeding outcomes or mortality
Mao et al., 2021 [194]	106 Chinese general surgery patients	Preoperative	TEG-MA, TEG R-time, TEG-K, TEG alpha-angle	DVT, hypercoagulability	TEG parameters were associated with DVT status and hypercoagulability
McCrath et al., 2005 [195]	240 US general surgery patients	Postoperative	TEG-MA	Complications	TEG-MA was predictive of thrombotic complications and myocardial infarction
Yamakage et al., 1998 [196]	30 Japanese general surgery patients	Intraoperative	TEGc	Coagulopathy	Celite-activated TEG parameters are predictive of coagulopathy
Bhattacharyya et al., 2021 [197]	50 critically ill Indian general surgery patients	Postoperative	TEG-MA, TEG R-time, TEG-K, TEG alpha-angle	Survival time, mortality	TEG parameters immediately postoperative were better predictors of survival than alternative measures
Ioscovich et al., 2016 [198]	22 Israeli general surgery patients with Gaucher disease	Preoperative	TEG-MA, TEG R-time, TEG-K, TEG alpha-angle	Hemostasis	TEG parameters may be useful in guiding treatment in this population
Ekin et al., 2019 [199]	77 Turkish reconstructive surgery patients	Preoperative, intraoperative, postoperative free flap reconstruction	TEG-MA, TEG R-time, TEG-K, TEG alpha-angle	Free flap loss	TEG parameters were not predictive of free flap loss
Zavlin et al., 2019 [200]	100 US reconstructive surgery patients	Preoperative, intraoperative, postoperative	TEG-MA, TEG R-time, TEG-K, TEG alpha-angle	Coagulopathy, thrombosis	TEG parameters predicted coagulopathy and thrombosis
Madsen et al., 2012 [201]	21 Danish maxillary patients	Intraoperative	TEG-MA, TEG R-time, TEG-K, TEG alpha-angle	Blood loss, platelet count	TEG parameters predicted blood loss and platelet count
Phillips et al., 2020 [202]	46 US neonatal ECMO patients	Intraoperative congenital diaphragmatic hernia surgery	TEG-guided anticoagulation vs. clinician discretion	Blood product usage	Introduction of TEG-guided anticoagulation protocol resulted in reduced blood product usage
Northrop et al., 2015 [203]	366 US pediatric ECMO patients	Intraoperative	TEG-guided anticoagulation protocol vs. clinician discretion	Blood product usage, hemorrhagic complications, ECMO circuit life	Introduction of TEG-guided anticoagulation protocol resulted in reduced blood product usage, decreased complications and increased ECMO circuit life
Moynihan et al., 2017 [204]	31 Australian pediatric ECMO patients	Intraoperative	TEG-MA, TEG R-time, TEG-K, TEG alpha-angle	Anticoagulation monitoring	TEG parameters are useful for intraoperative anticoagulation monitoring
Henderson et al., 2018 [205]	49 US pediatric ECMO patients	Intraoperative	TEG R-time	Hypocoagulation, thrombotic complications	TEG R-time was a predictor of thrombotic complication
Saini et al., 2016 [206]	46 US pediatric ECMO patients	Intraoperative	TEG-MA, TEG R-time, TEG-K, TEG alpha-angle	Bleeding complications	TEG parameters did not predict bleeding complications
Sleeper et al., 2021 [207]	40 US pediatric ECMO patients	Intraoperative	TEG-MA, TEG R-time, TEG-K, TEG alpha-angle	Bleeding events	TEG parameters are predictive of bleeding events
Alexander et al., 2010 [208]	27 Australian pediatric ECLS patients	Intraoperative	TEG kaolin and heperinase	aPTT, platelet count	TEG was a poor indicator of aPTT and an acceptable indicator of platelet count. Authors recommend using conventional laboratory tests in this population
Giani et al., 2021 [209]	25 Italian ECMO patients	Intraoperative	TEG R-time, ROTEM-INTEM	Anticoagulation monitoring	TEG R-time, ROTEM-INTEM, and conventional diagnostics had similar utility for monitoring anticoagulation status in ECMO
Panigada et al., 2016 [210]	32 Italian ECMO patients	Intraoperative	TEG “flat line” reading	Perioperative bleeding	TEG “flat line” was not related to bleeding outcomes
Ranucci et al., 2016 [211]	31 Italian ECMO patients	Intraoperative	TEG-MA, TEG R-time	Anticoagulation monitoring	TEG parameters are effective for intraoperative anticoagulation monitoring
Stammers et al., 1995 [212]	17 US ECMO patients	Intraoperative	TEG-MA, TEG R-time, TEG-K, TEG alpha-angle	Coagulopathy	TEG parameters predicted coagulopathy
Ziegler et al., 2008 [213]	49 Italian prostatectomy patients	Intraoperative, postoperative	TEG-LY30, TEG-LY40	Coagulopathy	TEG parameters did not predict postoperative coagulopathy
Bell et al., 1996 [214]	30 UK urology patients	Intraoperative and postoperative transurethral prostatectomy (TURP)	TEG clot lysis	Postoperative coagulation, blood loss	TEG clot lysis correlates with blood loss
Gal-Oz et al., 2020 [215]	417 Israeli renal biopsy patients	Intraoperative	TEG-MA	Bleeding time	TEG-MA did not predict bleeding time
Lu et al., 2020 [216]	713 Chinese renal patients	Intraoperative	TEG-MA, TEG R-time, TEG-K, TEG alpha-angle	Coagulopathy, VTE	TEG parameters were associated with coagulopathy and VTE and distinguished between patients with different renal diagnoses
Gatt et al., 2014 [217]	24 Maltese oncology patients	Preoperative and postoperative transfusion	TEG-MA, TEG R-time, TEG-K, TEG alpha-angle	Platelet count, coagulation	TEG parameters were predictive of platelet count
Moore et al., 2018 [218]	100 US oncology patients	Preoperative	TEG-MA, TEG R-time, TEG alpha-angle, TEG-LY30	Coagulopathy, tumor type, resection success	TEG parameters were associated with hypercoagulability, tumor type, and resection success
Wang et al., 2018 [219]	80 Chinese oncology patients	Intraoperative prostate malignancy resection	TEG-MA, TEG R-time, TEG-K, TEG alpha-angle	Postoperative bleeding	TEG parameters were predictive of postoperative bleeding
Lin et al., 2020 [220]	43 Chinese thoracic surgery patients	Preoperative, intraoperative	TEG-guided monitoring of intraoperative anticoagulation	Coagulopathy	TEG procedures were useful for monitoring anticoagulation status during surgery
Volod et al., 2017 [221]	98 US mechanical circulatory support device patients	Intraoperative	TEG-MA, TEG R-time, TEG-K, TEG alpha-angle	Anticoagulation monitoring	TEG parameters are useful for monitoring anticoagulation status

Strengths and limitations

To our knowledge, this is one of the reviews that addressed the application of TEG usage in monitoring the hematological outcomes in the perioperative periods including nearly all surgical procedures. Therefore, this review opens the doors for clinicians to reach out to recent evidence about TEG applications on patients having any surgery or procedure to enhance transfusion and coagulation-related management. In addition, we searched many databases and screened the relevant records in detail to include all relevant studies, which provide the recent updates in TEG applications in multiple surgeries.

The limitation is that this is only a literature review that summarizes existing research on TEG. It does not include other viscoelastic tests such as ROTEM. Most of the studies lacked comparison groups. While comparing with standardized laboratory tests, a controversy was observed between the related studies in the literature. In addition, lacking direct statistical analysis including all related studies made it difficult to solve the controversy about the efficacy of TEG usage in some surgeries. 

Summary

TEG showed promising results in detecting and improving hematological outcomes in patients who underwent major surgeries and procedures or who were critically ill; however, more comparative studies are needed to establish this efficacy. These promising results were observed in trauma surgeries regarding predicting mortality, hypercoagulability, and bleeding even when it was compared to conventional methods; however, its role to guide blood product transfusion was questionable. 

TEG was useful in monitoring anticoagulant therapy in orthopedics operations; however, its roles in predicting thrombotic events, hypercoagulability, or complications were questionable among the studies. The same controversy was observed in obstetric operations; however, it showed promising results in ICU patients, especially in the prediction or improvement of sepsis, coagulopathy, thrombotic events, ICU duration, hospital stay, and ventilator duration. 

In transplant surgeries, they effectively predicted hypercoagulation; however, their roles in predicting bleeding, blood product needs, and thrombotic events were still questionable. Regarding cardiovascular surgeries, they were effective in the prediction of the need for blood products, coagulopathy, and thrombotic events and they were effective in monitoring anticoagulation therapy. 

TEG parameters were useful in predicting coagulation and bleeding, preventing complications, and decreasing blood product transfusion in neurological surgeries; however, compared to the conventional tools, they were better in all these outcomes except for hypercoagulation, which had the same results. In abdominal surgeries, TEG was effective in bariatric, cystectomy, and gastric cancer surgeries; however, their results were controversial in hepatic, esophagectomy, and obstructive jaundice surgeries. The efficacy of TEG usage was also controversial in patients on ECMO whether they were adults or pediatrics. However, in general surgeries, a controversy was observed in pediatric patients while a promising efficacy was observed in adults regarding predicting hypercoagulation, thrombotic events, and blood product transfusion.

## Conclusions

Based on the evidence reviewed here we conclude that TEG can be used in a wide range of perioperative settings to guide transfusion and coagulation management and thereby influence certain outcomes. Because of some limitations addressed in this review, we recommend performing more randomized clinical trials comparing TEG parameters with standardized tools and performing meta-analyses to pool all related studies’ data to solve the controversy between studies. More clinical trials also are needed to investigate the usage of TEG in critically ill patients, especially in cardiothoracic, obstetric and oncology surgeries as well as patients on ECMO; geriatric and pediatric patients, and patients with renal disease. 
